# New developments in age-related macular degeneration

**Published:** 2008-09

**Authors:** Lyndon da Cruz

**Affiliations:** Consultant Ophthalmic Surgeon, Moorfields Eye Hospital, 162 City Road, London EC1V 2PD, UK.

**Figure:**
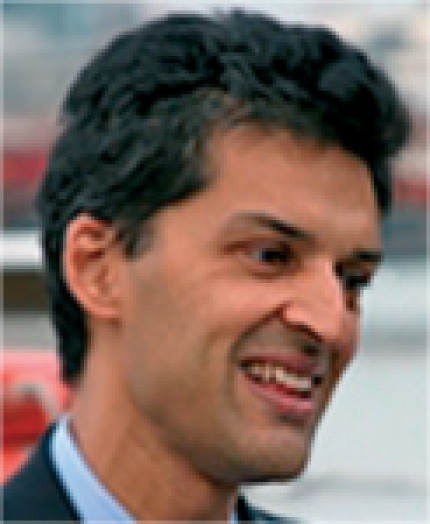


## Epidemiology and Classification

The World Health Organization (WHO) estimates that over 3 million people (9% of global blindness) are blinded by age-related macular degeneration (AMD). AMD affects people over the age of 55. There are two main types of AMD, dry and wet. In dry AMD, patients slowly lose vision through progressive atrophy of the macular tissue. Wet, or exudative, AMD, is associated with new blood vessels called subretinal neovascular membranes (or SRNVM) and affected patients lose vision more rapidly due to fluid leakage and haemorrhage at the macula.

## Recent developments in genetics

Originally, AMD was thought to be a degenerative disease with a genetic component, but strongly influenced by environmental factors, especially smoking. This changed in 2005, with the demonstration that a gene (complement factor H or CFH, a regulator of inflammation) is implicated in up to 50% of cases of AMD in some populations.^1^ This suggests that AMD may be associated with inflammatory processes rather than primarily being an environmentally determined degenerative disease.

Since then, other genes, predominantly associated with inflammation, have been linked to AMD: some increase the risk of AMD and others protect against it. These genes are markers of risk for both wet and dry AMD. Their prevalence varies between different ethnic groups.^2^

It is still not understood why an individual at risk will manifest dry or wet AMD; at present, the only known trigger for wet AMD is smoking – an environmental factor.

## New treatments for SRNVM in wet AMD

Although, there is still no effective therapy for dry AMD, new and more effective treatments for wet AMD have been developed.

Initial treatment protocols for SRNVM were restricted to thermal laser^3^ and, subsequently, photodynamic therapy (PDT).^4^ Though thermal laser was cheap to deliver, few patients were eligible for treatment and recurrence after laser was common. PDT allowed more patients to be treated, but the usual outcome was delayed vision loss rather than visual improvement.

The identification of the crucial role played by vascular endothelial growth factor (VEGF) in the pathogenesis of wet AMD has allowed the development of VEGF-blocking agents such as bevacizumab (Avastin), pegaptanib (Macugen) and ranibizumab (Lucentis).^5^ This is the first generation of drugs which start to control the vision loss from late disease in all types of wet AMD and, in some cases, to produce improvement.^6^

Macugen was shown to be more effective than PDT in treating wet AMD and to reduce the probability of developing severe vision loss (by about 50% over one year). However, like PDT, it only delayed vision loss but did not prevent it.

Subsequently, it was shown that Lucentis was more effective than Macugen and could prevent further vision loss: about 95% of patients maintained their baseline vision whilst on treatment. Furthermore, one third of patients experienced a gain of vision (more than 15 letters of visual acuity).

In 2005, it was demonstrated that Avastin, the molecule from which Lucentis is derived, could also be used for the treatment of wet AMD,^7^ although this was not its original use. Since then, the use of Avastin for the treatment of AMD has been widely reported; the literature suggests that its efficacy is similar to that of Lucentis, as well as its toxicity. The advantages of Avastin are a lower cost and longer half-life (allowing for six-weekly dosing, rather than four-weekly as for Lucentis).

**Figure:**
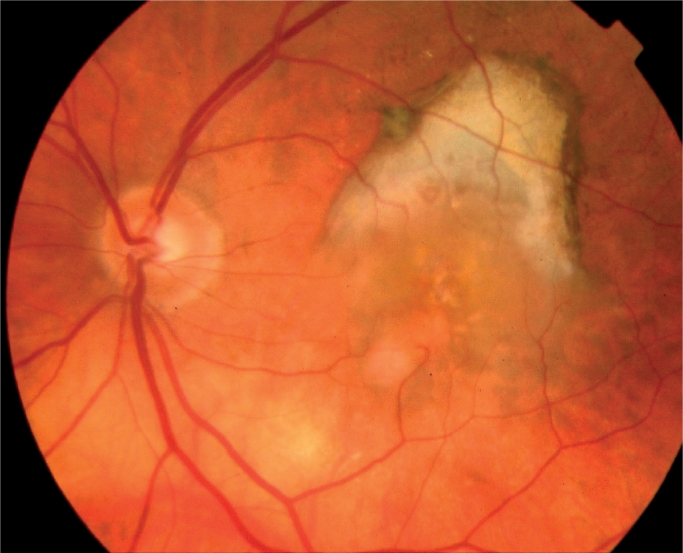
Wet AMD

## Combination treatment protocols

Possibly the most important recent addition to treatment protocols for wet AMD is the combination of two, or even three, modalities delivered simultaneously to minimise re-treatments and optimise gain in visual acuity. Intravitreal anti-VEGF agents have been combined with intravitreal steroids (either dexamethasone or triamcinolone) and, in some cases, in combination with PDT. This is called ‘triple therapy’. There are some reports suggesting that these triple treatments may reduce the need for re-treatment and may provide a ‘one shot’ treatment.

## Polypoidal chorio-vasculopathy

Recently, a subset of wet AMD, termed polypoidal chorio-vasculopathy (PCV), has been identified as a clinical entity.^8^ PCV is important, as it may be the main cause of vision loss from AMD in certain Asian and black populations.^8^ It has a different natural history, treatment, and prognosis relative to traditional wet AMD.

PCV is best demonstrated by ICG (indocyanine green) angiography, while wet AMD is shown with fluorescein. The distinctive clinical features of PCV include one or more haemorrhagic pigment epithelial detachments (PEDs), extensive exudation, and disease outside of the macula including the periphery. There is a predilection for peripapillary ‘polyps’. Patients are also characteristically younger.

Current treatments include: direct laser ablation of choroidal polyps^9^ seen on ICG angiography, PDT, and intravitreal anti-VEGF drugs – or a combination of all of these. There is no consensus regarding treatment protocol, but there are series reporting each modality as being effective. For a clinician, it is critical to suspect PCV from the clinical picture and the ethnicity of the patient, and then to investigate appropriately with ICG and identify the polyps for treatment.

## Conclusion

Over the last four years, age-related macular degeneration has become better understood and treatments have become much more effective. The demonstration of high-risk genes associated with the inflammation process has changed the approach to this disease. Also, the identification of VEGF as central to the pathogenesis of wet AMD has allowed the development of the first generation of drugs that start to control the vision loss from late disease. In the future, we may be able to identify at-risk individuals and, as well as stopping smoking, suggest other changes in lifestyle and perhaps offer long-term medication that can prevent the disease.
